# A van der Waals-like Transition Between Normal and Cancerous Phases in Cell Populations Dynamics of Colorectal Cancer

**DOI:** 10.1038/srep36620

**Published:** 2016-11-18

**Authors:** Kang Qiu, Li-fang Wang, Jian Shen, Alssadig A. M. Yousif, Peng He, Dan-dan Shao, Xiao-min Zhang, John B. Kirunda, Ya Jia

**Affiliations:** 1 Institute of Biophysics and Department of Physics, Central China Normal University, Wuhan 430079, China; 2 Department of Mathematics and Physics, Xuzhou Medical University, Xuzhou 221004, China

## Abstract

Based on a deterministic continuous model of cell populations dynamics in the colonic crypt and in colorectal cancer, we propose four combinations of feedback mechanisms in the differentiations from stem cells (SCs) to transit cells (TCs) and then to differentiated cells (DCs), the four combinations include the double linear (LL), the linear and saturating (LS), the saturating and linear (SL), and the double saturating (SS) feedbacks, respectively. The relative fluctuations of the population of SCs, TCs, and DCs around equilibrium states with four feedback mechanisms are studied by using the Langevin method. With the increasing of net growth rate of TCs, it is found that the Fano factors of TCs and DCs go to a peak in a transient phase, and then increase again to infinity in the cases of LS and SS feedbacks. The “up-down-up” characteristic on the Fano factor (like the van der Waals loop) demonstrates that there exists a transient phase between the normal and cancerous phases, our novel findings suggest that the mathematical model with LS or SS feedback might be better to elucidate the dynamics of a normal and abnormal (cancerous) phases.

Mathematical modeling used as an indispensable complementary tool has played an important role in understanding the biological processes and phenomena which cannot be explained directly through the experimental studies due to the limitations of the biological experimental techniques^1^. One such well-known example is to illustrate the cell population homeostasis in the intestinal crypt and the developmental processes of the colorectal cancer (CRC)^2^,^3^. It is widely believed that earlier the CRC is diagnosed, the longer the patient survives^4^. Given the opinion that the origins of CRC lie in crypts of the intestine, illustrating the mechanisms involved in the dynamics of cells in colonic crypts is essential to understand the development of CRC. Single colorectal crypt can be mainly subdivided into three compartments which contain the stem cells (SCs), transit cells (TCs) and differentiated cells (DCs) separately from the bottom to the top of the crypt. In a normal crypt, SCs with unlimited proliferative capacity can regulate the tissue renewal rate to maintain homeostasis, update the crypt totally every 4–5 days through generating the TCs which only undergo 4–6 times of division^5^,^6^, the TCs differentiate to DCs which exfoliate from the top of the crypt and form the luminal surface of gut. Johnston *et al*. proposed a deterministic compartmental continuous model^2^,^3^, which can elucidate the mechanisms involved in normal intestinal crypt homeostasis dynamics and the gradual stages of CRC, and the combinations of feedback mechanisms in their model were assumed in the differentiations from stem cells (SCs) to transit cells (TCs) and then to differentiated cells (DCs).

On the one hand, although the linear feedback mechanism can be used to illustrate the homeostasis in a normal crypt, it cannot explain the uncontrolled exponential growth of cell populations in an abnormal (cancerous) colonic crypt unless coincidentally mutations occur. Some researchers pointed out that the other factors, such as microenvironmental influences and abnormal regulation of signal pathway, can also induce the uncontrolled growth and the tumor initiation^7^,^8^,^9^,^10^,^11^,^12^. Thus, the saturating feedback mechanism, which was used to illustrate the uncontrolled cell population growth when the net growth rates of SCs or TCs are over a critical threshold value, may be better than the linear feedback in the continuous model^2^,^3^. Moreover, the feedback mechanism in the differentiation from SCs to TCs might not be same as that in the differentiation from TCs to DCs, the feedback in the differentiation from SCs to TCs maybe a linear or saturating, and that in the differentiation from TCs to DCs maybe a saturating or linear. Now a question is raised: which feedback mechanism is the better to elucidate the physiological properties in a normal and abnormal (cancerous) colonic crypt?

On the other hand, various experimental evidences suggested that the number of SCs in a normal colonic crypt is often very limited^13^,^14^,^15^,^16^,^17^,^18^. For instance, Potten and Loeffler concluded that different experimental techniques or assumptions got apparently different SCs numbers, the range of them is approximately 30–40, less than 16 or 4–16^13^. More following experiments demonstrated that the number is much smaller and is about 4–6 in each colonic crypt by using different experimental techniques^14^,^15^,^16^. More recently, Moore and Lyle found that the SCs number was only between 1.8 and 3.5 in human duodenum by using a two-dimensional model based on experimental results^17^. Kozar *et al*. revealed that the numbers of actual functional stem cells in intestinal crypts (5–7 in each crypt) and adenomas (9 per gland) are small through continuous clonal labeling^18^. The large relative fluctuations of cell populations due to the small number of SCs cannot be neglected in the growth of the three types of cells in intestinal crypt. Recently, based on the deterministic continuous model with two linear feedbacks, Pei *et al*.^19^ studied relative fluctuations of cell populations with the LL feedbacks. Thus, another question is raised: what are the diversities of relative fluctuations of cell populations around equilibrium states under the different combinations of feedback mechanisms?

In this paper, based on the deterministic model, we firstly use a Hill function form to represent a general feedback mechanism (including the linear and the saturating), then there are four combinations of feedback mechanisms in the cell populations dynamics, i.e. the LL, LS, SL, SS feedbacks. Secondly, in order to study the relative fluctuations in the populations of SCs, TCs, and DCs, we derive the formulae of Fano factor, covariance and susceptibility of cell populations around equilibrium states by using the Langevin method^20^. Our theoretical results show that the stationary populations of TCs and DCs exhibit an approximately threshold behavior as a function of the net growth rate of TCs for the four feedback mechanisms. The reproductions of TCs and DCs can be classified into three phases: normal, transient, and cancerous. It is interesting that there is a so-called van der Waals loop in the Fano factors of TCs and DCs populations under the LS and SS feedback mechanisms, which can be used to as evidence of a phase transition^21^,^22^,^23^,^24^. Our results demonstrate that LS and SS feedback mechanisms are more appropriate to illustrate the dynamics of the CRC, and the transient (coexistent) phase may correspond to the transient state (i.e., adenomas) before the CRC initiation^25^,^26^,^27^,^28^. Thirdly, we compare our theoretical results with these obtained by Gillespie algorithm^29^ (an accurately simulating). We end with the conclusions and discussions.

## General model with four feedback mechanisms

**Figure 1:**
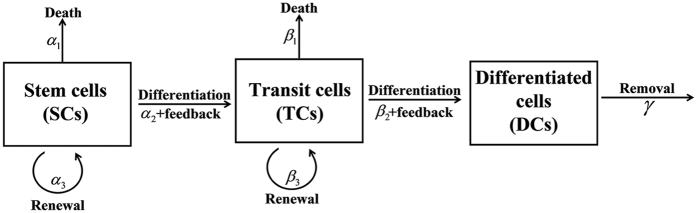
Schematic diagram of the three compartments (SCs, TCs, and DCs) in a single crypt. The SCs differentiate into TCs which in turn differentiate into DCs, where the both differentiation rates *α*_2_ and *β*_2_ are controlled by feedbacks. SCs and TCs can also renew with rates *α*_3_ and *β*_3_, respectively, and die with rates *α*_1_ and *β*_1_, respectively. DCs can only die or be removed out of the compartment with rate *γ*.

A general deterministic continuous model, which is based on the three compartments assumption in a single crypt^30^ and can illustrate the homeostasis of the three types of cells (SCs, TCs, and DCs) in a colonic crypt and tumor initiation of a CRC^2^,^3^, is used to describe the cell populations dynamics in a colonic crypt. The schematic representation of these three compartments in a crypt is given by Fig. 1 2.

In order to make the system structurally stable, the differentiations of both SCs and TCs are regulated by two alternative forms of feedback which maintain homeostasis in the crypt, respectively. Here we use a Hill function form to represent two possible feedback mechanisms (linear or saturating):


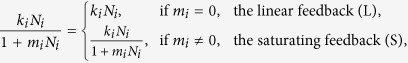


where *i* = 0 and 1, *N*_0_ and *N*_1_ are the population numbers of SCs and TCs, respectively, *k*_0_ and *k*_1_ represent the rates of population response to change, while *m*_0_ and *m*_1_ are feedback parameters. In the case of saturating feedback, there is a saturating value *α*_2_ + *k*_0_/*m*_0_ (or *β*_2_ + *k*_1_/*m*_1_) of the differentiation rate of SCs (or TCs). Therefore, there are four possible combinations (listed in Table 1), that is, LL feedback (*m*_0_ = 0 and *m*_1_ = 0), LS feedback (*m*_0_ = 0 and *m*_1_ = 0.01), SL feedback (*m*_0_ = 0.1 and *m*_1_ = 0), and SS feedback (*m*_0_ = 0.1 and *m*_1_ = 0.01).

**Figure 2:**
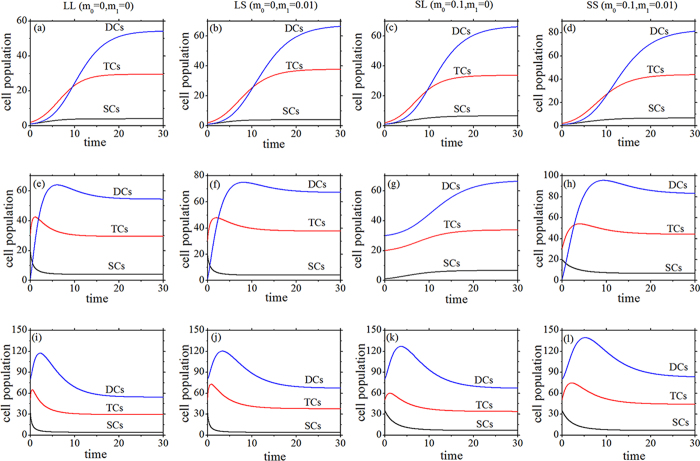
Time course of SCs, TCs, and DCs with different feedbacks and initial conditions. Initial conditions: upper row (**a–d**) *N*_0_(0) = 1.0, *N*_1_(0) = 1.0, and *N*_2_(0) = 1.0; middle row (**e–h**) *N*_0_(0) = 20.0, *N*_1_(0) = 30.0, and *N*_2_(0) = 1.0; bottom row (**i–l**) *N*_0_(0) = 35.0, *N*_1_(0) = 50.0, and *N*_2_(0) = 80.0. The left parameter values are *α* = 0.4, *β* = 0.2, and *α*_2_ = 0.3. All the parameters are measured in hr^−1^.

In the deterministic description, the population dynamics in the crypt can be given by following ordinary differentiated equations (ODEs)^2^,^3^


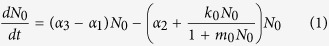



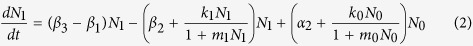



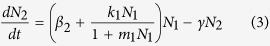


which give the steady state


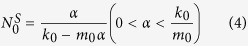



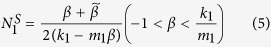



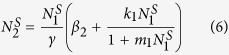


with


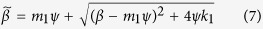



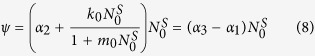


and *N*_2_ is the number of DCs, and *α* = *α*_3_ − *α*_2_ − *α*_1_ and *β* = *β*_3_ − *β*_2_ − *β*_1_, which denote the net (per capita) growth rates of SCs and TCs, respectively. Combining that *α*_1_ + *α*_2_ + *α*_3_ = 1 and *β*_1_ + *β*_2_ + *β*_3_ = 1, the net growth rates (*α* and *β*) can be determined by the linear stability analysis: 0 < *α* < *k*_0_/*m*_0_ and −1 < *β* < *k*_1_/*m*_1_. So the *k*_0_/*m*_0_ and *k*_1_/*m*_1_ are the critical threshold values which discriminate the non-cancerous and cancerous states. When one of the net growth rates goes beyond its own threshold value, the cell population grows uncontrolled. In this paper, the values of the parameters *β*_2_ = 0.3, *γ* = 0.323, *k*_0_ = 0.1 and *k*_1_ = 0.01 are determined^2^,^3^. All the four situations can satisfy the healthy physiological case that the number of SCs is small and the number of each type of cells (SCs, TCs, and DCs) can get a stationary value under different initial conditions of cell population as time goes on (Fig. 2). Above results demonstrate that all the four feedbacks can control the cell population growth and make the system maintains homeostasis.

In the stochastic description, the dynamics of the three compartments are given by the following stochastic equations:


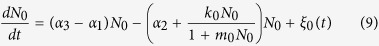



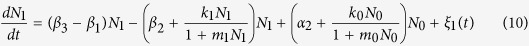



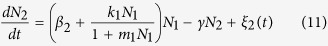


where *ξ*_*i*_(*t*) (*i* = 0, 1, 2) are random variables. Here, the statistical properties of the random variables *ξ*_*i*_(*t*) around the steady state are derived by using two assumptions^20^ (see Part 1 of Supplementary Information). All the mean values of *ξ*_*i*_(*t*) are zero, but the autocorrelation functions of *ξ*_*i*_(*t*) relate with the system’s intrinsic characters (the parameters and the steady values of the system) and can be described by following equations


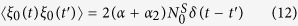



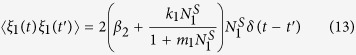



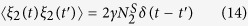


and the cross-correlations between *ξ*_*i*_(*t*) take the forms


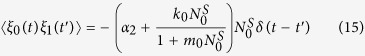



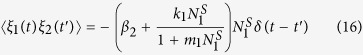



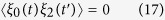


From the above equations (12)–(17), 34, 34, 34, 34, 34, it is found that the autocorrelation of *ξ*_0_(*t*) is determined by the SCs’s net growth rate *α*, differentiation rate *α*_2_, and its steady state value 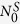, while the autocorrelation of *ξ*_1_(*t*), the cross-correlation between *ξ*_0_(*t*) and *ξ*_1_(*t*), and the cross-correlation between *ξ*_1_(*t*) and *ξ*_2_(*t*) have connection with the feedback mechanism on SCs or TCs. The autocorrelation of *ξ*_2_(*t*) is dependent of DCs’s steady state value and the removal rate. There is no cross-correlation between *ξ*_0_(*t*) and *ξ*_2_(*t*).

## Results

### Relative fluctuation formulae of cell populations around equilibrium states

Equations (9)–(11), 34, 34 can be linearized around the steady state (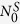, 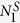, 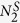) with 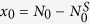, 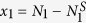 and 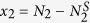. By virtue of equations (4)–(8), 10, 10, 10, 10, equations (9)–(11), 34, 34 can be rewritten as


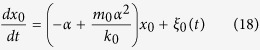



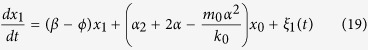



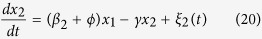


where


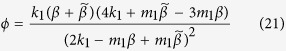


The corresponding Fokker-Planck equation of the equations (18)–(20), 27, 28 is^31^,^32^


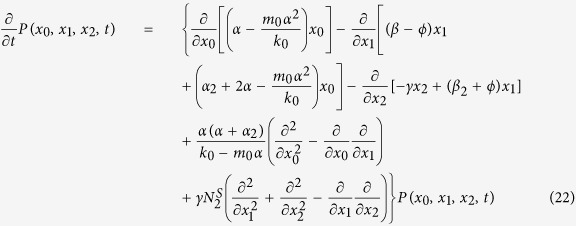


According to the definition of the Fano factor (the ratio of the variance to the steady state value), it is used to characterize the relative size of fluctuation and can be calculated through the Fokker-Planck equation. So, we can get the formulae of Fano factors of SCs, TCs, and DCs, respectively


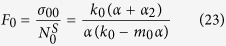



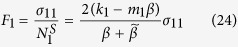



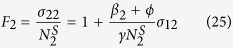


where the variances (*σ*_00_, *σ*_11_, *σ*_22_) of three compartments and the covariance (*σ*_01_, *σ*_02_, *σ*_12_), which can describe the correlations between fluctuations of the cell population, are given in Part 2 of Supplementary Information.

In addition, the susceptibility (which measures the sensitivity to variation of a parameter *z*^33^,^34^) can be calculated by using its definition form


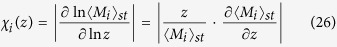


where 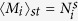
(*i* = 0, 1, 2), which means the steady value of the system. Here, we consider the effects of the net growth rates, so *z* = *α, β*. According to this definition, the susceptibilities to the two net growth rates are derived in Part 3 of Supplementary Information.

### Comparisons the diversities with four feedbacks due to the net growth rates

Although all the four feedbacks can maintain the homeostasis of the population in the colonic crypt (Fig. 2), the mutational processes, steady state values, and Fano factors are quite different under different feedbacks due to the net growth rates (*α* and *β*). Next, the differences on the mutational processes, steady state values, and the diversities of the fluctuation effects on Fano factors due to the net growth rates (*α* and *β*) of SCs and TCs in a colonic crypt with four feedback mechanisms are discussed in the range of physiologically reasonable parameter values^2^,^3^.

#### A. Mutational processes and tumor initiation

**Figure 3:**
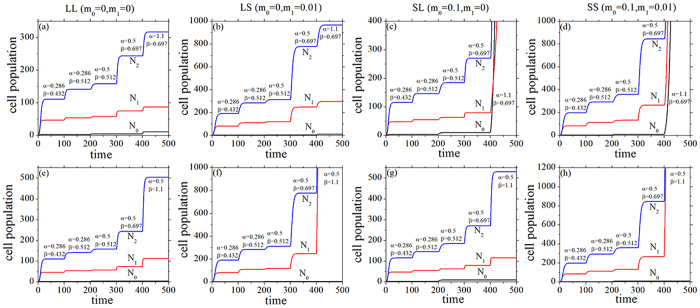
Time course of SCs, TCs, and DCs in a sequence of mutations with different feedbacks. The initial parameters are taken to be *N*_0_(0) = 1.0, *N*_1_(0) = 1.0, *N*_2_(0) = 1.0, and *α* = 0.286, *β* = 0.432, *α*_2_ = 0.3, *β*_2_ = 0.3. The mutations cause, successively, *β* = 0.512, *α* = 0.5, *β* = 0.697^3^. The last mutation is *α* = 1.1 in the upper row, and *β* = 1.1 in the bottom row. All the parameters are measured in hr^−1^.

According to equations (1)–(3), 10, 10, the mutational processes with four cases of feedbacks are illustrated in Fig. 3. Here, we consider the mutations which can increase the net growth rate *α* or *β*, and the mutations can give a selective advantage to the cell^2^,^3^. The same initial parameter set *N*_0_(0) = 1.0, *N*_1_(0) = 1.0, *N*_2_(0) = 1.0, and *α* = 0.286, *β* = 0.432, *α*_2_ = 0.3, *β*_2_ = 0.3 was chosen with the four cases of feedbacks. As time goes on, the processes go with the same sequence of mutations in all the four feedbacks except the last mutation in which *α* = 1.1, *β* = 0.697 (upper row of Fig. 3) or *α* = 0.5, *β* = 1.1 (bottom row of Fig. 3). Above two situations denote that the last mutation can cause an extreme large selective advantage, i.e. the net growth rate *α* is beyond the threshold value *k*_0_/*m*_0_ or *β* is beyond the threshold value *k*_1_/*m*_1_. It is obvious that the total population of the crypt increases gradually with four feedbacks, which can illustrate a gradual stages with the mutational processes. However, there always have limited steady state values and the dynamics of the colonic crypt is controllable with LL feedback (first column of Fig. 3). When a saturating feedback is included, such as LS (second column of Fig. 3), SL (third column of Fig. 3), and SS (fourth column of Fig. 3) feedbacks, there can result in an uncontrolled growth of the cell population with the net growth rate beyond the threshold value of the saturating feedback (Fig. 3(c,d,f,h)). Thus, the model with one or two saturating feedbacks is appropriate to illustrate the processes of tumor initiation.

#### B. Steady states

**Figure 4:**
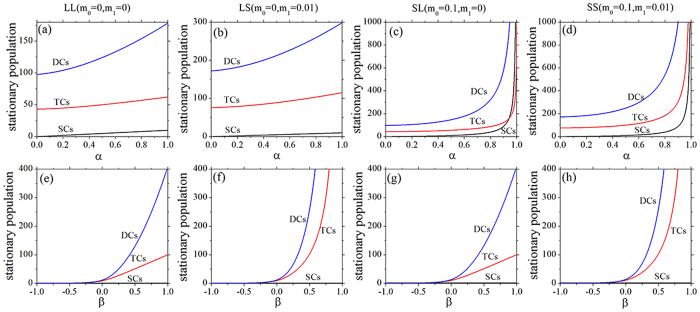
Stationary population as a function of net growth rates *α* (the upper row) and *β* (the bottom row). (**a–d**) *α*_2_ = 0.2 and *β* = 0.432. (**e–h**) *α* = 0.2 and *α*_2_ = 0.3. All the parameters are measured in hr^−1^.

From equations (4)–(6), 10, 10 and above results, the steady state values of SCs, TCs, and DCs are dependent on the systemic parameters. Here, we study the effects of one single net growth rate (*α* or *β*) on the steady state values, and the cell population as a function of *α* or *β* with four feedbacks is shown by Fig. 4. For a given value of *β*, with the increasing of *α*, the stationary population of SCs is small and linearly increased with LL and LS feedbacks (Fig. 4(a,b)), but it is linearly increased first, then to a uncontrolled infinite value later with SL and SS feedbacks (Fig. 4(c,d)). At the same time, the cell population of TCs or DCs increases nonlinearly with all the four feedbacks (Fig. 4(a–d)), and is controllable with LL and LS feedbacks (Fig. 4(a,b)), but uncontrollable with SL and SS feedbacks (Fig. 4(c,d)). It can conclude that, when the net growth rate *α* of SCs is close to the saturating threshold (*k*_0_/*m*_0_ = 1) with SL and SS feedback, the population of the crypt is rapidly increased and the tumor is more likely to be initiated. According to equation (4), when the feedback on the SCs is linear (*m*_0_ = 0), i.e. LL and LS feedbacks, the stationary population of SCs is *α*/*k*_0_ which is small because of *k*_0_ = 0.1 and is proportional to *α*(0 ≤ *α* ≤ 1); when the feedback on the SCs is saturating (*m*_0_ = 0.1), i.e. SL and SS feedbacks, the stationary population of SCs is 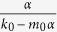 which becomes uncontrollable with *α* close to the saturating threshold (*k*_0_/*m*_0_ = 1).

For a given value of *α*, with the increasing of *β*, the stationary population of SCs is a constant (see equation (4)) and small (Fig. 4(e–h)), the stationary populations of TCs and DCs exhibit an approximately threshold behavior, and the threshold is around *β* = 0 with all the four feedbacks (Fig. 4(e–h)). However, when the net growth rate *β* is larger than 1 (the threshold value), the whole population of the crypt is still controlled and limited with LL and SL feedbacks (Fig. 4(e,g)). Above results show that only when feedback on TCs is saturating can the uncontrolled increasing of cell populations or the cancerous phase be described, e.g. the LS or SS feedback, the population of the crypt increases uncontrolled and the cancerous phase are shown by Fig. 4(f,h). In the cases of LS or SS feedback, the development of the colonic crypt can be classified into three phases: normal (*β* ≪ 0), transient (*β* ~ 0), and cancerous (*β* ≫ 0)^19^, which can also correspond to three phases of tumor evolution: breakthrough, expansion and invasion^35^.

#### C. A van der Waals-like Loop on Fano Factors

**Figure 5:**
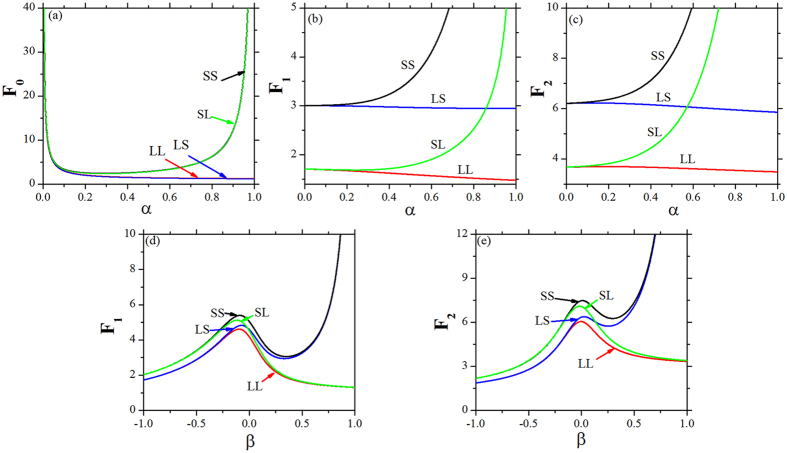
Diversities of fluctuation effects on Fano factors due to net growth rates with four different feedbacks. (**a–c**) *α*_2_ = 0.2, *β* = 0.432. (**d,e**) *α* = 0.2, *α*_2_ = 0.3. All the parameters are measured in hr^−1^.

Above theoretical results indicate that the number of SCs in a crypt is always small, which is consistent with the experimental data in normal colonic crypt, except the SL and SS feedbacks with larger net growth rate *α* (Fig. 4(c,d)). Due to the small number of SCs, the relative high fluctuations of populations which can be characterized by the Fano factor are studied around the steady state by Langevin method. The Fano factors of SCs, TCs, and DCs with four feedbacks as a function of *α* are illustrated by Fig. 5(a–c) sequentially. With a fixed net growth rate *β (β* = 0.432), it is obvious that the three Fano factors (*F*_0_, *F*_1_, *F*_2_) with SL and SS feedbacks are quite different with LL and LS feedbacks. With the increasing of *α*, the Fano factor of SCs goes down rapidly for small values of *α* with four feedbacks, then reaches 1 for large values of *α* with LL and LS feedbacks, but goes up quickly again to infinity with SL and SS feedbacks when *α* is close to 1 (the threshold value *k*_0_/*m*_0_) (Fig. 5(a)); the Fano factors of TCs and DCs decrease linearly and slowly with LL and LS feedbacks but increase nonlinearly and quickly to infinity with SL and SS feedbacks (Fig. 5(b,c)). These effects of the net growth rate of SCs on the relative fluctuation around the steady state can be understood directly. When the net growth rate *α* is small, the numbers of SCs is very small (Fig. 4(a–d)) and the noise is relatively large on the SCs. When *α* increases, the cell population of SCs will increase and the relative intrinsic fluctuation goes down (Fig. 5(a)). Due to considering the effects of the net growth rate *α*, which puts effects directly on the SCs, the type of feedback on SCs determines the variation tendency of Fano factors (*F*_0_, *F*_1_, *F*_2_) (Fig. 5(a–c)). When *α* is close to the threshold value 1 with SL and SS feedbacks, the numbers of SCs, TCs, and DCs increase uncontrolled to infinity (Fig. 4(c,d)), then the Fano factors also increase to infinity (Fig. 5(a–c)).

Fano factors of TCs and DCs with four feedbacks as a function of *β* with determined *α* are shown by Fig. 5(d,e), while the Fano factor of SCs which has no relation with *β* (see equation (23)) is a constant. With the increasing of *β*, the Fano factors of both TCs and DCs with four feedbacks increase first, reach a maximum, and then decrease to a constant with LL and SL feedbacks but again increase uncontrolled to infinity with LS and SS feedbacks (Fig. 5(d,e)). When *β* < 0, the variation tendency of Fano factors is controlled by the type of feedback on SCs and on TCs when *β* > 0. The peak of Fano factors of TCs or DCs occurs in the transient phase (*β* ~ 0) (Fig. 5(d,e)), which means that there is a significant change of relative fluctuations caused by an abruptly enhanced cell population of TCs or DCs. When *β* is close to the threshold value (*k*_1_/*m*_1_ = 1) with the LS or SS feedback, the numbers of TCs and DCs increase uncontrolled to infinity (Fig. 4(f,h)) and the Fano factors also go up to infinity (Fig. 5(d,e)). This “up-down-up” characteristic on the Fano factors is similar to the van der Waals loop (Fig. 5(d,e)) which corresponds to the ‘PV-isotherm’ in the van der Waals theory of phase transition. This loop gives a continuous transition from liquid to vapor or from vapor to liquid, and it can demonstrate that there is a metastable state (superheated liquid or supercooled vapor) during this process. Thus, this van der Waals-like loop demonstrate that there is a transient phase between the normal and cancerous phases^21^,^22^,^23^,^24^ in this paper. It was demonstrated that there is a transient benign state (e.g., adenomas)^25^,^26^,^27^,^28^ or an abrupt increase of TCs^36^,^37^,^38^ before CRC initiation or expansion phase of other tumors initiation^35^.

**Figure 6:**
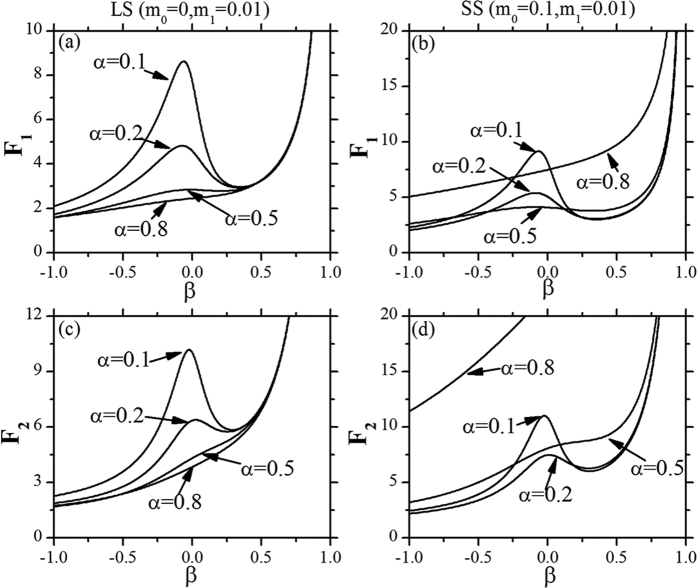
Diversities of fluctuation effects on Fano factors of TCs and DCs due to *β* with different *α*. *α*_2_ = 0.3. All the parameters are measured in hr^−1^.

Figure 6 shows the diversities of fluctuation effects on Fano factors of TCs and DCs due to *β* with different *α* under LS and SS feedbacks respectively. For the LS feedback, with the increasing of *α*, the intensity of relative fluctuations goes down in the normal (*β* ≪ 0) and transient (*β* ~ 0) phases and is almost same in the cancerous phase (*β* ≫ 0 or *β* > 0.5) (Fig. 6(a,c)). This is because that the Fano factors of TCs and DCs (*F*_1_ and *F*_2_) is decreasing with the increasing of *α* under the LS feedback (Fig. 5(b,c)), but when *β* > 0.5, i.e. in the cancerous phase, the *β* is the dominate factor, and the intensity of relative fluctuations is almost same. For the SS feedback, with the increasing of *α*, the behavior of Fano factors is different. When *α* is small (*α* < 0.5), the intensity of relative fluctuations goes down with the increasing of *α* in the normal and transient phases and vice vesa (Fig. 6(b,d)). In the cancerous phase, the Fano factors *F*_1_ and *F*_2_ go to the infinity with different *α*. From Fig. 5(b,c), it is obvious that *F*_1_ and *F*_2_ increase nonlinearly and quickly with the increasing of *α* except for small *α* under the SS feedback. Except these differences between LS and SS feedbacks, there are some common points with these two feedbacks. There always exists a van der Waals loop with small *α* with the increasing of *β* (Fig. 6), which means there is a transient phase during the expansion of the tumor. However, when *α* is large, the tumor will go directly from normal phase to cancerous phase, just like the sublimation from liquid to vapor at high temperature.

In addition, the effects of the net growth rate *α* or *β* on the covariance and susceptibilities are compared and provided in Part 4 and 5 of Supplementary Information.

### Comparisons the results with different methods

The aim of this paper is mainly to compare the effects of the relative fluctuations of the cell population in a colonic crypt around the steady state with four feedbacks. There are mainly three methods to study the fluctuations of biochemical reaction systems. The first one is to solve the probability distribution for all the molecular components through the chemical master equation^39^, which can be solved explicitly only in rare cases. In most of cases, the probability distribution is given out through computer simulation methods, such as the Gillespie algorithm^29^. The second technique is to use the linear noise approximation (LNA) to approximately solve the master equation which is simplified to a linear Fokker-Planck equation by using van Kampen’s expansion^39^. The third approach is the Langevin method^20^ which is mainly used in this paper. This method is more intuitive than the Langevin theory (the second method) and these two methods are equivalent at steady state^20^. Here, we consider the additive noises as the relative fluctuation sources of the cell population in a colonic crypt (see equations (9)–(11), 34, 34).

Comparing the Langevin method of Swain^20^ with the usual Langevin theory or LNA^39^, the statistical properties of random variables, such as mean square value, self-correlation function and mutual correlation function, are dependent of its own systemic parameters and the steady state values, and can be derived through two assumptions (i.e. the one-step biochemical processes and the small fluctuation at the steady state) in the Langevin method^20^, whereas the usual Langevin theory^20^ only artificially gives the statistical properties of intrinsic noises.

**Figure 7:**
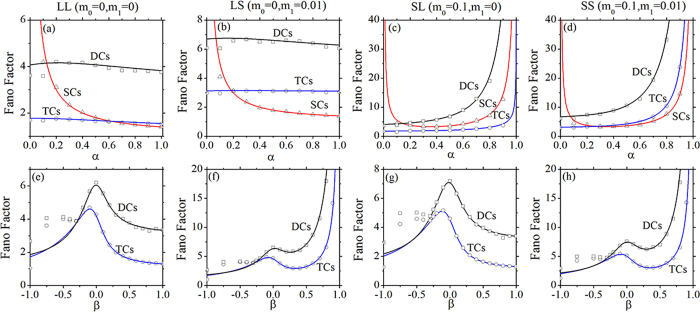
Fano factors as a function of *α* (upper row) and *β* (bottom row) obtained by Langevin method^20^ (lines) and Gillespie algorithm^29^ (symbols) with Ω = 200. (**a–d**) *α*_2_ = 0.4, *β* = 0.432. (**e–h**) *α* = 0.2, *α*_2_ = 0.3. All the parameters are measured in hr^−1^.

**Figure 8:**
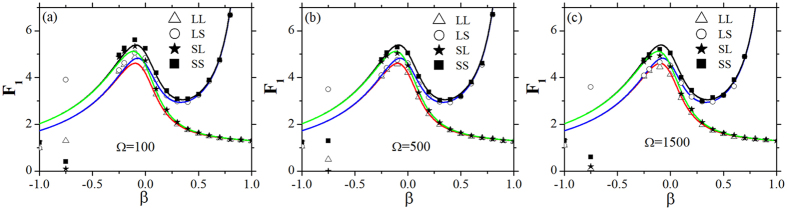
Fano factors of TCs as a function of *β* obtained by Langevin method^20^(lines) and Gillespie algorithm^29^ (symbols) with LL, LS, SL and SS feedbacks with different Ω. (**a–c**) *α* = 0.2, *α*_2_ = 0.3. All the parameters are measured in hr^−1^.

Comparing the results of the Langevin method^20^ with those of Gillespie algorithm (an accurate simulating)^29^, the theoretical and simulation results of the Fano factors as a function of *α* and *β* are compared with the same and different volumes of parameter Ω in Figs 7 and 8 with four feedbacks, respectively. It is obvious that the two kinds of results are consistent with each other in most region of parameters *α* and *β* except about 0 ≤ *α* < 0.1 and −1 ≤ *β* < −0.4. Because the number of the populations of SCs is very small with low values of net growth rates of *α* and *β* with four feedbacks (see Fig. 4), the relative intrinsic fluctuations will be very high, which is against the second assumption of the Langevin method (the small fluctuation at the steady state), and then the divergence between the results of the two approaches is remarkable. However, the results of the Fano factors around the transient phase (the peaks) are consistent with each other by using of the two methods (Figs 7 and 8). So, our theoretical results obtained by the Langevin method are valid with large enough number of populations, i.e. 0.1 < *α* < 1 and −0.4 < *β* < 1.

## Conclusions and Discussions

In this paper, we start from a general model of the colonic crypt cell population dynamics which contains four combinations of feedback mechanisms, i.e. LL, LS, SL and SS. Based on this general deterministic continuous model, the relative fluctuations between the populations of SCs, TCs, and DCs are considered, and the statistical properties of fluctuations are derived from the system’s own systemic parameters and steady state values by using the two assumptions of Langevin method^20^.

The formulae of Fano factors, covariances, and susceptibilities are derived with the general model. Although the dynamics of cell populations of SCs, TCs, and DCs can reach a steady state with the four feedbacks, which is reasonable in the range of physiologically parameter values, the steady state values, and the effects of relative fluctuations on Fano factors, covariances, and susceptibilities obtained by using of Langevin method^20^ are different as a function of the net growth rate *α* (or *β*) of SCs (or TCs). Our theoretical results show that the stationary populations of TCs and DCs exhibit an approximately threshold behavior as a function of the net growth rate of TCs for the four feedback mechanisms. The reproductions of TCs and DCs can be classified into three phases: normal, transient, and cancerous. The LL feedback which cannot produce the uncontrolled growth is not suitable for the cancerous phase with increasing of the net growth rate of SCs or TCs. It is interesting that there is a so-called van der Waals loop in the Fano factors of TCs and DCs populations under the LS and SS feedback mechanisms, which can be considered as an evidence of phase transition, and this transient phase may be corresponded to the adenomas state before CRC tumor initiation^25^,^26^,^27^,^28^ or expansion phase in other tumors initiation^35^.

This three-compartment model discussed in this paper is a general model in normal tissue differentiation or the development of other tumors initiation. Thus, it is important to know which one is the best of these four feedback mechanisms for modeling the dynamics of a normal and abnormal (CRC) colonic crypt or other tumors initiation? Due to the difficulty of identifying cancer stem cells (CSCs), some researchers believed that the quantity of CSCs is small in tumors, just like the number of SCs in the normal tissues. A recent research^18^ reports that CSCs serve only to replace stem cell loss, with rare clonal victors driving gland repopulation and tumor growth, and the number of CSCs per crypt is five to seven in CRC. Our results show that the LL and LS feedbacks are better than the other two, which always can control the number of SCs in a small value. The LL feedback cannot illustrate the uncontrolled cell populations growth unless a coincidentally irreversible genetic mutation happens to remove the one of the two linear feedbacks. However, a recent report revealed that chronic myeloid leukemia can be cured by decreasing the proliferating rate of TCs without killing CSCs^40^, which means the cancerous phase can be reversed to the normal phase with controlling the net growth rate of TCs. Therefore, the LS feedback is better than LL feedback. On the other hand, some experiments demonstrate that the CSCs number can be a substantial proportion of the tumor^41^,^42^,^43^, such as in melanoma^41^, some human acute myeloid leukemia (AML)^42^ or in more aggressive type or late stage of cancer, e.g. breast cancer^43^. Then these may use distinct mechanisms from other solid malignancies^44^, and the SL and SS feedbacks may be more appropriate to illustrate this situation, because the SCs number can be large and any proportion when the net growth rate *α* increases. However, above theoretical results and the other results^2^,^3^,^18^,^36^,^37^,^38^ showed that the net growth rate of TCs plays an important role in the tumor initiation and metastasis. Thus, we suggest that the feedback on the TCs is probable a saturating feedback in a real colonic crypt and in colorectal cancer or in other tumors. In a conclusion, the saturating feedback on the TCs is necessary and the feedback on the SCs may be linear or saturating depending on the stages or types of the tumor.

## Additional Information

**How to cite this article**: Qiu, K. *et al*. A van der Waals-like Transition Between Normal and Cancerous Phases in Cell Populations Dynamics of Colorectal Cancer. *Sci. Rep.* **6**, 36620; doi: 10.1038/srep36620 (2016).

**Publisher’s note**: Springer Nature remains neutral with regard to jurisdictional claims in published maps and institutional affiliations.

## Supplementary Material

Supplementary Information

## Figures and Tables

**Table 1:** Combinations of feedback mechanisms.

Feedback Parameters	*m*_1_ = 0	*m*_1_ = 0.01
*m*_0_ = 0	**LL feedback**	**LS feedback**
*m*_0_ = 0.1	**SL feedback**	**SS feedback**
