# Supplementary material for: A van der Waals-like Transition Between Normal and Cancerous Phases in Cell Populations Dynamics of Colorectal Cancer
Source: Sci Rep. 2016 Nov 18;6:36620. doi: 10.1038/srep36620 (PMC5114675; doi:10.1038/srep36620)
Supplement: Supplementary Information [file srep36620-s1.doc]

**Supplementary Information**

**A van der Waals-like Transition Between** **Normal and Cancerous Phases in Cell Populations Dynamics of Colorectal Cancer**

Kang Qiu^1,2^, Li-fang Wang^1^, Jian Shen^1^, Alssadig A. M. Yousif^1^, Peng He^1^, Dan-dan Shao^1^, Xiao-min Zhang^1^, John B. Kirunda^1^, and Ya Jia^1*^

^1^ *Institute of Biophysics and Department of Physics, Central China Normal University, Wuhan 430079, China*

^2^ *Department of Mathematics and Physics, Xuzhou Medical University, Xuzhou 221004, China*

*Correspondence and requests for materials should be addressed to Y. J. (email: [jiay@phy.ccnu.edu.cn](mailto:jiay@phy.ccnu.edu.cn))

**1. Two assumptions of Langevin method**

Using the two assumptions of Langevin method^1^, the statistical properties of the random variables $\xi_{i}(t)$ in equations (9)-(11) (in main text) at the steady state ($N_{0}^{S}$,$N_{1}^{S}$, and $N_{2}^{S}$) can be derived.

**Assumption (i):** one-step biochemical processes. The time interval $\delta t$ should be small enough that only one event can occur, and $\xi_{i}(t)\delta t$ ($i=0,1,2$) can only be $\pm1$ or zero. The probability $P(u,v,w)$ is denoted as $\xi_{0}(t)\delta t=u$, $\xi_{1}(t)\delta t=v$, $\xi_{2}(t)\delta t=w$. At the steady state and from equations (9)-(11) (in main text), we have $P(1,0,0)=(\alpha_{3}-\alpha_{1})N_{0}^{S}\delta t$, $P(-1,1,0)=(\alpha_{2}+\frac{k_{0}N_{0}^{S}}{1+m_{0}N_{0}^{S}})N_{0}^{S}\delta t$, $P(0,1,0)=(\beta_{3}-\beta_{1})N_{1}^{S}\delta t$, $P(0,0,-1)=\gamma N_{2}^{S}\delta t$, $P(0,-1,1)=(\beta_{2}+\frac{k_{1}N_{1}^{S}}{1+m_{1}N_{1}^{S}})N_{1}^{S}\delta t$, and the other $P(u,v,w)$ equal zero. According to these conditions, the mean of random variables $\xi_{i}(t)$ at steady state obeys

$\left\langle\xi_{0}(t)\delta t\right\rangle=P(1,0,0)\times1+P(-1,1,0)\times(-1)=0$ (S1)

$\left\langle\xi_{1}(t)\delta t\right\rangle=P(-1,1,0)\times1+P(0,1,0)\times1+P(0,-1,1)\times(-1)=0$ (S2)

$\left\langle\xi_{2}(t)\delta t\right\rangle=P(0,-1,1)\times1+P(0,0,-1)\times(-1)=0$ (S3)

and the mean square at steady state satisfies

$\left\langle\xi_{0}(t)^{2}\delta t^{2}\right\rangle=2(\alpha+\alpha_{2})N_{0}^{S}\delta t$ (S4)

$\left\langle\xi_{1}(t)^{2}\delta t^{2}\right\rangle=2(\beta_{2}+\frac{k_{1}N_{1}^{S}}{1+m_{1}N_{1}^{S}})N_{1}^{S}\delta t$ (S5)

$\left\langle\xi_{2}(t)^{2}\delta t^{2}\right\rangle=2\gamma N_{2}^{S}\delta t$ (S6)

**Assumption (ii):** the small fluctuation at the steady state. The deviation caused by the relative fluctuations is always small compared to the steady values. Then, it can be considered that there is no correlation between $\xi_{i}(t)$ and $\xi_{i}(t')$ for all . So, the self-correlations and mutual correlations of the random variables can be mathematically described by the $\delta$ functions (equations (S7)-(S12)).

$\left\langle\xi_{0}(t)\xi_{0}(t'\right\rangle\langle\rangle=2(\alpha+\alpha_{2})N_{0}^{S}\delta(t-t'$ (S7)

$\left\langle\xi_{1}(t)\xi_{1}(t'\right\rangle\langle\rangle=2(\beta_{2}+\frac{k_{1}N_{1}^{S}}{1+m_{1}N_{1}^{S}})N_{1}^{S}\delta(t-t'$ (S8)

$\left\langle\xi_{2}(t)\xi_{2}(t'\right\rangle\langle\rangle=2\gamma N_{2}^{S}\delta(t-t'$ (S9)

$\left\langle\xi_{0}(t)\xi_{1}(t'\right\rangle\langle\rangle=-(\alpha_{2}+\frac{k_{0}N_{0}^{S}}{1+m_{0}N_{0}^{S}})N_{0}^{S}\delta(t-t'$ (S10)

$\left\langle\xi_{1}(t)\xi_{2}(t'\right\rangle\langle\rangle=-(\beta_{2}+\frac{k_{1}N_{1}^{S}}{1+m_{1}N_{1}^{S}})N_{1}^{S}\delta(t-t'$ (S11)

$\left\langle\xi_{0}(t)\xi_{2}(t'\right\rangle\langle\rangle=0$ (S12)

**2. Formulae of variances and covariances**

The moments of $x_{i}(t)$ $(i=0,1,2)$ are defined by

$\frac{d\left\langle x_{i}^{n}\right\rangle}{dt}=\int\int\int_{-\infty}^{+\infty} x_{i}^{n}\frac{\partial}{\partial t}P(x_{0},x_{1},x_{2},t)dx_{0}dx_{1}dx_{2}$ (S13)

and using the normalization condition of $P(u,v,w)$,

$\int\int\int_{-\infty}^{+\infty} P(x_{0},x_{1},x_{2},t)dx_{0}dx_{1}dx_{2}=1$ (S14)

and the definition of mean, we can get

$\left\langle x_{i}^{n}\right\rangle=\int\int\int_{-\infty}^{+\infty} x_{i}^{n}P(x_{0},x_{1},x_{2},t)dx_{0}dx_{1}dx_{2}$ (S15)

where $P(u,v,w)$ satisfies^2^

$\lim_{x_{i}\to\pm\infty} P(x_{0},x_{1},x_{2},t)=0$ (S16)

$\lim_{x_{i}\to\pm\infty} \frac{\partial}{\partial x_{j}}P(x_{0},x_{1},x_{2},t)=0,for\begin{matrix} & i,j=0,1,2. \end{matrix}$ (S17)

By using the Fokker-Planck equation (equation (22) in main text) and equations (S13)-(S17), we have

$\frac{d\left\langle x_{0}^{2}\right\rangle}{dt}=-2(\alpha-\frac{m_{0}\alpha^{2}}{k_{0}})\left\langle x_{0}^{2}\right\rangle+\frac{2\alpha(\alpha+\alpha_{2})}{k_{0}-m_{0}\alpha}$ (S18)

$\frac{d\left\langle x_{1}^{2}\right\rangle}{dt}=2(\beta-\varphi)\left\langle x_{1}^{2}\right\rangle+2(\alpha_{2}+2\alpha-\frac{m_{0}\alpha^{2}}{k_{0}})\left\langle x_{0}x_{1}\right\rangle+2\gamma N_{2}^{S}$ (S19)

$\frac{d\left\langle x_{2}^{2}\right\rangle}{dt}=-2\gamma\left\langle x_{2}^{2}\right\rangle+2(\beta_{2}+\varphi)\left\langle x_{1}x_{2}\right\rangle+2\gamma N_{2}^{S}$ (S20)

$\frac{d\left\langle x_{0}x_{1}\right\rangle}{dt}=(\alpha_{2}+2\alpha-\frac{m_{0}\alpha^{2}}{k_{0}})\left\langle x_{0}^{2}\right\rangle+\text{（}\beta-\varphi-\alpha+\frac{m_{0}\alpha^{2}}{k_{0}})\left\langle x_{0}x_{1}\right\rangle-\frac{\alpha(\alpha+\alpha_{2})}{k_{0}-m_{0}\alpha}$ (S21)

$\frac{d\left\langle x_{0}x_{2}\right\rangle}{dt}=\text{（}\beta_{2}+\varphi)\left\langle x_{0}x_{1}\right\rangle-(\alpha+\gamma-\frac{m_{0}\alpha^{2}}{k_{0}})\left\langle x_{0}x_{2}\right\rangle$ (S22)

$\frac{d\left\langle x_{1}x_{2}\right\rangle}{dt}=\text{（}\beta_{2}+\varphi)\left\langle x_{1}^{2}\right\rangle+(\alpha_{2}+2\alpha-\frac{m_{0}\alpha^{2}}{k_{0}})\left\langle x_{0}x_{2}\right\rangle+(\beta-\gamma-\varphi)\left\langle x_{1}x_{2}\right\rangle-\gamma N_{2}^{S}$ (S23)

At the steady state, equations (S18)-(S23) can be written in a matrix equation:

$\mathbf{A\Xi}+\mathbf{B}=0$ (S24)

where

$\mathbf{A}=\left(\begin{matrix} -2(\alpha-\frac{m_{0}\alpha^{2}}{k_{0}}) & 0 & 0 & 0 & 0 & 0 \\ 0 & 2(\beta-\varphi) & 0 & 2(\alpha_{2}+2\alpha-\frac{m_{0}\alpha^{2}}{k_{0}}) & 0 & 0 \\ 0 & 0 & -2\gamma& 0 & 0 & 2(\beta_{2}+\varphi) \\ \alpha_{2}+2\alpha-\frac{m_{0}\alpha^{2}}{k_{0}} & 0 & 0 & \beta-\varphi-\alpha+\frac{m_{0}\alpha^{2}}{k_{0}} & 0 & 0 \\ 0 & 0 & 0 & \beta_{2}+\varphi& -(\alpha+\gamma-\frac{m_{0}\alpha^{2}}{k_{0}}) & 0 \\ 0 & \beta_{2}+\varphi& 0 & 0 & \alpha_{2}+2\alpha-\frac{m_{0}\alpha^{2}}{k_{0}} & \beta-\gamma-\varphi\end{matrix}\right)$ (S25)

$\mathbf{\Xi}^{T}=(\sigma_{00},\sigma_{11},\sigma_{22},\sigma_{01},\sigma_{02},\sigma_{12})$ (S26)

$\mathbf{B}^{T}=\left(\frac{2\alpha(\alpha+\alpha_{2})}{k_{0}-m_{0}\alpha},2\gamma N_{2}^{S},2\gamma N_{2}^{S},-\frac{\alpha(\alpha+\alpha_{2})}{k_{0}-m_{0}\alpha},0,-\gamma N_{2}^{S}\right)$ (S27)

Solve the equation (S24)

$\sigma_{00}=\frac{k_{0}(\alpha+\alpha_{2})}{(k_{0}-m_{0}\alpha)^{2}}$ (S28)

$\sigma_{01}=\frac{k_{0}(\alpha+\alpha_{2})}{[k_{0}(\varphi+\alpha-\beta)-m_{0}\alpha^{2}]}\sigma_{00}$ (S29)

$\sigma_{11}=\frac{\gamma}{\varphi-\beta}N_{2}^{S}+\frac{2k_{0}\alpha+k_{0}\alpha_{2}-m_{0}\alpha^{2}}{k_{0}(\varphi-\beta)}\sigma_{01}$ (S30)

$\sigma_{02}=\frac{k_{0}(\beta_{2}+\varphi)}{k_{0}\alpha+k_{0}\gamma-m_{0}\alpha^{2}}\sigma_{01}$ (S31)

$\sigma_{12}=\frac{1}{\gamma+\varphi-\beta}\left[(\beta_{2}+\varphi)\sigma_{11}+(\alpha_{2}+2\alpha-\frac{m_{0}\alpha^{2}}{k_{0}})\sigma_{02}-\gamma N_{2}^{S}\right]$ (S32)

$\sigma_{22}=N_{2}^{S}+\frac{\beta_{2}+\varphi}{\gamma}\sigma_{12}$ (S33)

**3. Formulae of susceptibilities**

According to the definition of the susceptibility,

$\chi_{i}(z)=\left|\frac{\partial\ln\left\langle M_{i}\right\rangle_{st}}{\partial\ln z}\right|=\left|\frac{z}{\left\langle M_{i}\right\rangle_{st}}\cdot\frac{\partial\left\langle M_{i}\right\rangle_{st}}{\partial z}\right|$ (S34)

where $\left\langle M_{i}\right\rangle_{st}=N_{i}^{s}$ $(i=0,1,2)$, which means the steady value of the system. Here, we consider the effects of the net growth rates, so $z=\alpha,\beta$. Thus, the susceptibilities as a function of the two net growth rates ($\alpha$ and $\beta$) are derived

$\chi_{0}(\alpha)=1+\frac{m_{0}\alpha}{k_{0}-m_{0}\alpha}$ (S35)

$\chi_{1}(\alpha)=\left|\frac{\alpha}{N_{1}^{S}}\cdot\frac{\partial N_{1}^{S}}{\partial\alpha}\right|$ (S36)

$\chi_{2}(\alpha)=\left|\frac{\alpha}{N_{2}^{S}}\cdot\frac{\partial N_{2}^{S}}{\partial\alpha}\right|$ (S37)

$\chi_{0}(\beta)=0$ (S38)

$\chi_{1}(\beta)=\left|\frac{\beta}{N_{1}^{S}}\cdot\frac{\partial N_{1}^{S}}{\partial\beta}\right|$ (S39)

$\chi_{2}(\beta)=\left|\frac{\beta}{N_{2}^{S}}\cdot\frac{\partial N_{2}^{S}}{\partial\beta}\right|$ (S40)

where

$\frac{\partial N_{2}^{S}}{\partial\alpha}=\frac{1}{\gamma}(\beta_{2}+\frac{k_{1}N_{1}^{S}(m_{1}N_{1}^{S}+2)}{(1+m_{1}N_{1}^{S})^{2}})\frac{\partial N_{1}^{S}}{\partial\alpha}$ (S41)

$\frac{\partial N_{1}^{S}}{\partial\alpha}=\frac{\alpha}{2(k_{1}-m_{1}\beta)}(m_{1}+\frac{2k_{1}+m_{1}^{2}\phi-m_{1}\beta}{\sqrt{(\beta-m_{1}\phi)^{2}+4k_{1}\phi}})\frac{\partial\phi}{\partial\alpha}$ (S42)

$\frac{\partial\phi}{\partial\alpha}=\left[\alpha_{2}+\frac{k_{0}N_{0}^{S}(m_{0}N_{0}^{S}+2)}{(1+m_{0}N_{0}^{S})^{2}}\right]\frac{\partial N_{0}^{S}}{\partial\alpha}$ (S43)

$\frac{\partial N_{0}^{S}}{\partial\alpha}=\frac{k_{0}}{(k_{0}-m_{0}\alpha)^{2}}$ (S44)

$\frac{\partial N_{2}^{S}}{\partial\beta}=\frac{1}{\gamma}\left[\beta_{2}+\frac{k_{1}N_{1}^{S}(m_{1}N_{1}^{S}+2)}{(1+m_{1}N_{1}^{S})^{2}}\right]\frac{\partial N_{1}^{S}}{\partial\beta}$ (S45)

(S46)

Zero value of the susceptibility $\chi_{0}(\beta)$ (equation (S38)) means that the net growth rate of TCs can not influence the stationary population of SCs which only depend on its own net growth rate (see equation (4) in main text). However, other susceptibilities ($\chi_{2}$ and $\chi_{3}$) for the stationary populations of TCs and DCs are dependent on not only the net growth rates $\alpha$and $\beta$ but also their steady state values.

**4. Diversities of fluctuation effects on covariances**

Fig. S1 shows the covariances between the cell populations as a function of $\alpha$ or $\beta$ with the LL, LS, SL, and SS feedbacks. The covariance can measure how much the two compartments change together. For a given $\beta$ and with the increasing of $\alpha$ (Figs. S1(a)-S1(c)), it is obvious that the variation tendency of LL and LS feedbacks (or SL and SS feedbacks) is same because of the same type of feedback on the SCs. The covariance $\sigma_{01}$ is linearly increased with LL and LS feedbacks because of the linear feedback between SCs and TCs compartments, but it is nonlinearly increased with SL and SS feedbacks because of the saturating feedback between SCs and TCs compartments (Fig. S1(a)); however, $\sigma_{02}$ is nonlinearly increased even with LL feedback because the SCs and DCs compartments are not neighbor compartments (Fig. S1(b)); the covariance $\sigma_{12}$ is linearly increased with LL feedback, but nonlinearly increased with LS, SL, and SS feedbacks (Fig. S1(c)). Besides, the SL and SS feedbacks can induce extremely large values of the three covariances with $\alpha\text{\textasciitilde}0.5$, which means that the saturating feedback is more sensitive to the net growth rate $\alpha$ than the linear feedback, whereas the three covariances always have limited values with LL and LS feedback due to the linear feedback between SCs and TCs compartments.

For a given $\alpha$ and with the increasing of $\beta$ (Figs. S1(d)-S1(f)), when $\beta<0$, the variation tendency of the three covariances is controlled by the type of feedback on SCs and on TCs when $\beta>0$. There is a peak value of the variance $\sigma_{01}$ around the transient phase ($\beta\text{\textasciitilde}0$) (Fig. S1(d)), just like the effects of net growth rate $\beta$ on the Fano factors (Figs. 5(d) and 5(e) in main text), and $\sigma_{01}$ increases again to infinity with SS feedback and to a limited value with LS feedback (Fig.S1(d)); the covariance $\sigma_{02}$ between SCs and DCs is nonlinearly increased to a saturating value with LL and SL feedbacks, to a limited value with LS feedback and to an infinite value with SS feedback, and there is an abrupt increase around the transient phase (Fig. S1(e)); $\sigma_{12}$ is nonlinearly increased to a limited value with LL and SL feedbacks after an abrupt increase around the transient phase and it will go up to infinity with LS and SS feedbacks with $\beta$ close to the threshold value 1 because the feedback between TCs and DCs is a saturating feedback (Fig. S1(f)).


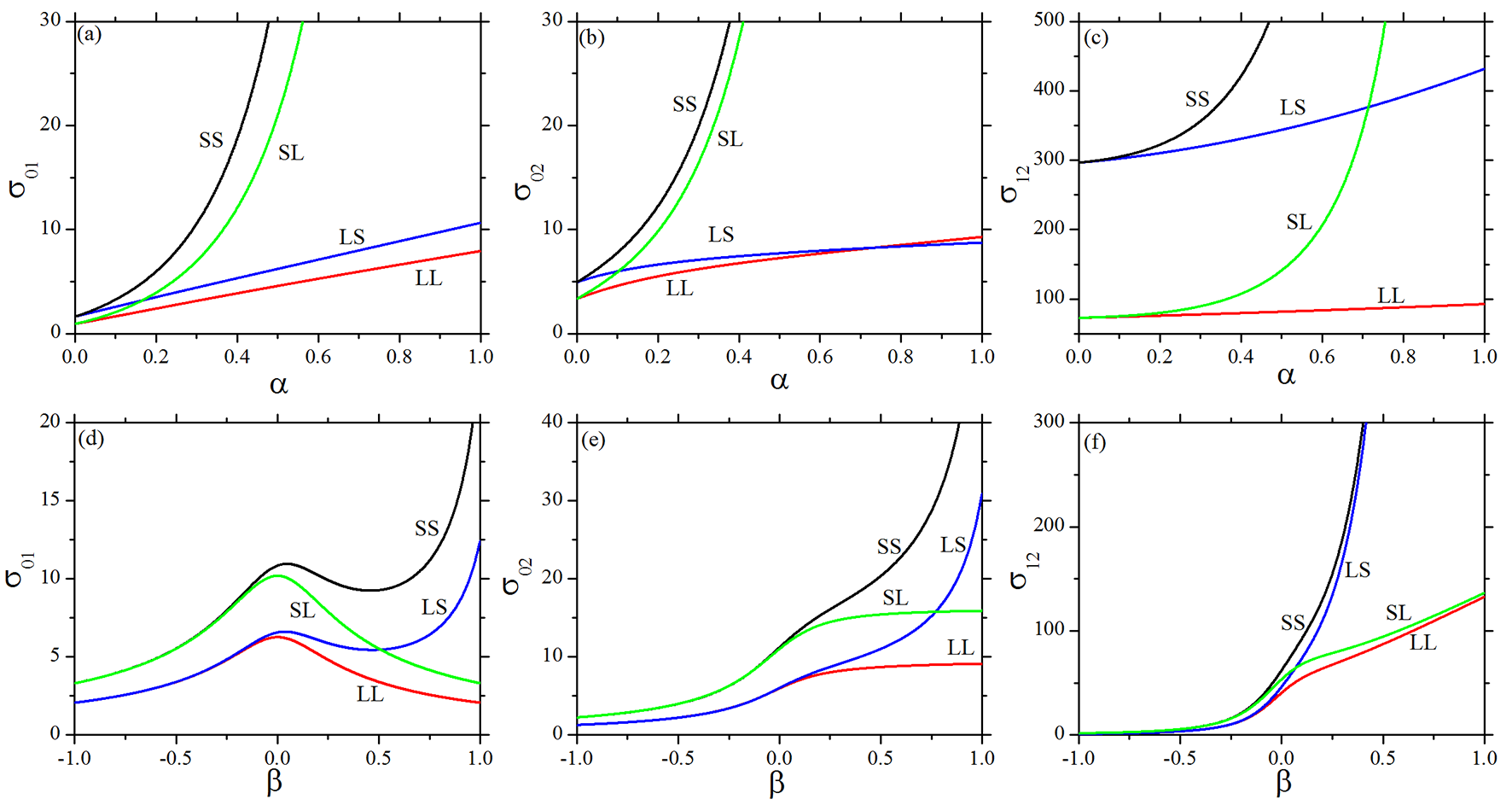
**Figure S1. Diversities of fluctuation effects on Fano factors due to net growth rates with the four different feedbacks.** (a)-(c): $\alpha_{2}=0.2$, $\beta=0.432$. (d)-(f): $\alpha=0.2$, $\alpha_{2}=0.3$. All the parameters are measured in hr^-1^.

**5. Diversities of fluctuation effects on susceptibilities**

The susceptibilities of the stationary populations of TCs and DCs as a function of the parameter $\alpha$ or $\beta$ are shown by Fig. S2. It is obvious that the susceptibility of SCs to $\beta$ is zero (see equation (S38)) with four feedbacks and is 1 to $\alpha$ with LL and LS feedbacks (Fig. S2(a)) where $m_{0}=0$(see equation (S35)). It means that the stationary population of SCs is not sensitive to the variation of parameters $\beta$ with all the four feedbacks and $\alpha$with LL and LS feedbacks. However, with the SL and SS feedbacks, the susceptibility of SCs to $\alpha$ is nonlinearly increased to infinity (Fig. S2(a)).

For given values of $\beta$ and $\alpha_{2}$, the susceptibilities of TCs and DCs are increased with all four feedbacks, but faster with SL and SS feedbacks than with the other two (Figs. S2(b) and S2(c)). For given values of $\alpha$ and $\alpha_{2}$, Figs.S2(d) and S2(e) show that when $\beta<0$, the variation tendency of the susceptibilities is almost same with the four feedbacks and is controlled by the type of feedback on TCs when $\beta>0$. The susceptibilities of TCs and DCs to the parameter $\beta$ decrease to zero at $\beta=0$ which is around the transient phase, and then increase to infinity with LS and SS feedbacks and to a saturating value with LL and SL feedbacks.


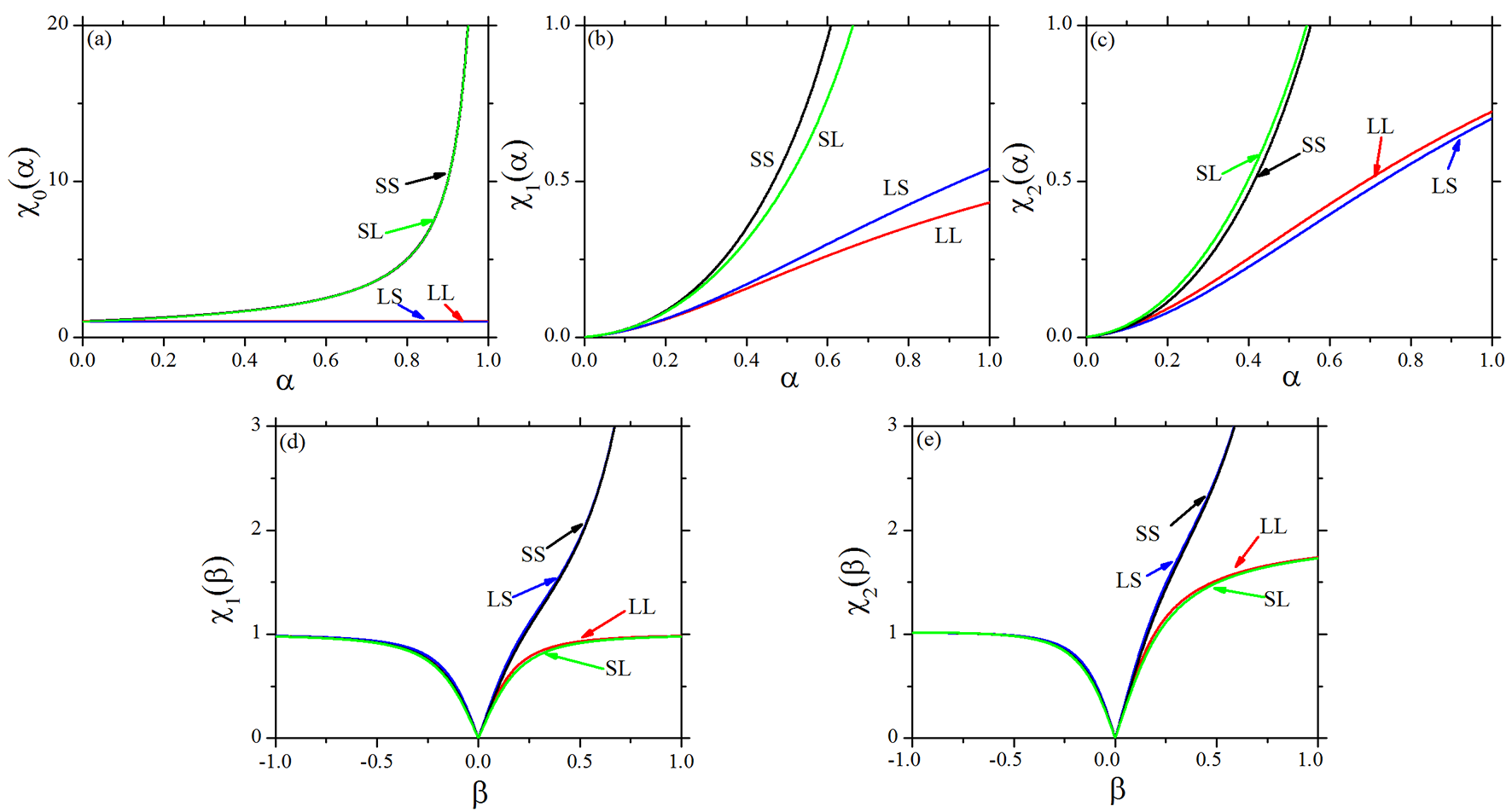
**Figure S2. Diversities of fluctuation effects on Fano factors due to net growth rates with the four different feedbacks.** (a)-(c): $\alpha_{2}=0.2$, $\beta=0.432$. (d)-(e): $\alpha=0.2$, $\alpha_{2}=0.3$. All the parameters are measured in hr^-1^.

**References**

1. Swain, P. S. Efficient attenuation of stochasticity in gene expression through post-transcriptional control. *J. Mol. Biol.* **344,** 965-976 (2004).

2. Tao, Y. Intrinsic noise, gene regulation and steady-state statistics in a two-gene network. *J. Theor. Biol.* **231,** 563-568 (2004).
